# The effect of preheated versus room-temperature skin disinfection on bacterial colonization during pacemaker device implantation: a randomized controlled non-inferiority trial

**DOI:** 10.1186/s13756-015-0084-1

**Published:** 2015-11-03

**Authors:** Camilla Wistrand, Bo Söderquist, Anders Magnusson, Ulrica Nilsson

**Affiliations:** Faculty of Medicine and Health, School of Health Sciences, Örebro, SE 701 82 Sweden; Faculty of Medicine and Health, School of Medical Sciences, Örebro, SE 701 82 Sweden; Clinical Epidemiology and Biostatistics, Faculty of Medicine and Health, Örebro University, Örebro, SE 701 82 Sweden; Department of Cardiothoracic surgery and Vascular surgery, University hospital in Örebro, Grevrosengatan, 701 85 Örebro, Sweden

**Keywords:** Perioperative, Skin disinfection, Bacterial growth, Non-inferiority

## Abstract

**Background:**

In clinical practice, patients who are awake often comment that cold surgical skin disinfectant is unpleasant. This is not only a problem of patients’ experience; heat loss during the disinfection process is a problem that can result in hypothermia. Evidence for the efficacy of preheated disinfection is scarce.

We tested whether preheated skin disinfectant was non-inferior to room-temperature skin disinfectant on reducing bacterial colonization during pacemaker implantation.

**Methods:**

This randomized, controlled, non-inferiority trial included 220 patients allocated to skin disinfection with preheated (36 °C) or room-temperature (20 °C) chlorhexidine solution in 70 % ethanol. Cultures were obtained by swabbing at 4 time-points; 1) before skin disinfection (skin surface), 2) after skin disinfection (skin surface), 3) after the incision (subcutaneously in the wound), and 4) before suturing (subcutaneously in the wound).

**Results:**

The absolute difference in growth between patients treated with preheated versus room-temperature skin disinfectant was zero (90 % CI −0.101 to 0.101; preheated: 30 of 105 [28.6 %] vs. room-temperature: 32 of 112 [28.6 %]). The pre-specified margin for statistical non-inferiority in the protocol was set at 10 % for the preheated disinfectant. There were no significant differences between groups regarding SSIs three month postoperatively, which occurred in 0.9 % (1 of 108) treated with preheated and 1.8 % (2 of 112) treated with room-temperature skin disinfectant.

**Conclusion:**

Preheated skin disinfection is non-inferior to room-temperature disinfection in bacterial reduction. We therefore suggest that preheated skin disinfection become routine in clean surgery.

**Trial registration:**

The study is registered at ClinicalTrials.gov (NCTO2260479).

## Background

Health-care-associated infections are a global concern for patient safety [1, 2]. With emerging antibiotic resistance, it is important to find safe preventive measures [3–5].

During clean surgery, such as pacemaker implantation, surgical site infections (SSIs) are a rare (1–1.25 %) but serious complication [6–8]. Pathogens isolated from SSIs are mainly staphylococci, both *Staphylococcus aureus* and coagulase-negative staphylococci, and streptococci [1, 4, 9]. Studies have shown that reducing the number of contaminating bacteria can prevent SSIs [10, 11]. Bacteria causing SSIs originate from the patient or the surgical team [12, 13].

Skin disinfection reduces the number of bacteria, thereby reducing SSIs [1]. According to the Cochrane Collaboration, there is insufficient research regarding the effects of skin disinfection [14]. In clinical practice, patients comment on the chill they experience during skin disinfection prior to surgery. This is not only a problem of patients’ comfort; heat loss during the disinfection procedure can cause hypothermia [15]. Hypothermia causes complications including myocardial events, SSIs, coagulopathy, and prolonged hospitalization [16, 17]. A pilot study showed that preheated disinfectant seemed to be comparable to room-temperature disinfectant in reducing bacterial growth [18]. To our knowledge there are no other studies reported that have examined the effectiveness of preheated skin disinfectant on bacterial colonization or SSIs.

The primary aim of this study was to test if preheated (36 °C) skin disinfectant is non-inferior to room-temperature (20 °C) skin disinfectant regarding skin colonization. The secondary aim was to investigate whether gender had an impact on differences in bacterial colonization in the surgical wound or SSIs among patients undergoing surgery.

## Methods

### Study design and participants

This study was a randomized, controlled, non-inferiority trial that included 220 patients undergoing pacemaker, implantable cardioverter-defibrillator, or cardiac resynchronization therapy under local anaesthesia. The study was performed at a cardiothoracic and vascular surgery department in Sweden. Inclusion criteria were age 18 years or older and ability to read and understand Swedish. Exclusion criteria were infection in an existing implanted device. The Regional Ethical Review Board of Uppsala approved the study (reference number 2012/255). Written informed consent was obtained.

### Intervention and randomization

Patients were included consecutively after arriving in the operating room (OR). Enrolment to the operation was done by an external controller who had no knowledge of the present study. Patients were randomly allocated to skin disinfectant solution (chlorhexidine 5 mg/mL in 70 % ethanol, Fresenius Kabi AS, Halden, Norway) that was preheated or at room temperature. Allocation took place directly after patients provided informed consent. Patients were stratified by gender and randomly allocated based on a computer-generated randomization list made by an independent statistician. The patient and the laboratory technician that performed the analysis were blinded to the allocation.

### Data collection

Patients showered twice with Descutan®, a 4 % chlorhexidine soap (Fresenius Kabi AB, Uppsala, Sweden), prior to surgery. Most patients received elective surgery and arrived at the hospital on the morning of surgery. Following standard procedures, intravenously administered antibiotic prophylaxis (cloxacillin 2 g) was given in the ward 15–30 min prior to surgery. The operating room had an average temperature of 19 °C with upward displacement ventilation. Sterile disposable surgical gowns and indicator gloves were worn by the OR team. Participants underwent skin disinfection during 2 min. The skin disinfectant was stored at room-temperature and kept at 20 °C, while the preheated skin disinfectant was stored in a warming cupboard and kept at 36 °C. The manufacturer provided a written statement that the bottles could be stored in a warming cupboard at temperatures up to 40 °C for 7 days without changing the compound.

The participants were disinfected from the cheek down and over the sternum according to routine procedures. Sterile draping was for single use only. Cultures were obtained at four time-points using a nylon-flocked swab (ESwab, COPAN Italia S.p.A., via Perotti 10, Brescia, Italy); 1) before skin disinfection on the skin surface, 2) after skin disinfection on the skin surface, 3) directly after the incision (subcutaneously in the wound), and 4) before closing with sutures (subcutaneously in the wound). Swabs for cultures were moistened with saline then rubbed for 15 s on the skin surface (incision site, approximately 10 mm × 50 mm). Swabs taken in the wounds were rubbed along the inside of the incision and along the edges for 15 s with a dry swab. Surgery was performed by a cardiologist. Cultures were kept cold until their arrival at the laboratory then analysed according to a specific study protocol.

The swabs were vortexed for a few seconds and 50 μl aliquots of the liquor transportation media was subcultured in hematin agar medium 4.3 % (w/v) (Columbia Blood Agar Base, Acumedia Neogen Corporation, Lansing, MI, USA) supplemented with 6 % (v/v) chocolatized defibrinated horse blood and incubated at 36 °C under aerobic conditions.. Samples were also subcultured on FAA plates (LAB 90 Fastidious Anaerobe Agar 4.6 % [w/v]; LAB M Ltd., Lancashire, UK) supplemented with 5 % (v/v) defibrinated horse blood and incubated under anaerobic conditions (10 % H_2_, 10 % CO_2_, 80 % N_2_) at 37 °C for 5 days. After 24 and 48 h of incubation under aerobic conditions or 5 days under anaerobic conditions, bacterial growth was determined quantitatively (colony forming units [cfu]/mL). Culture diagnostics and species verification were performed based on characteristic colony morphology, and using routine diagnostic procedures.

### Patient follow-up

After surgery, all patients were followed-up for three months to detect SSIs, which were defined according to the United States Centers for Disease Control and Prevention (CDC) criteria for SSI [1].

### Statistical analyses

Analyses were performed using SPSS, version 22. The primary outcome was based on a non-inferiority hypothesis, and the sample size was guided by an earlier study [18]. A sample size of 102 participants per group provided 80 % power at a one-sided significance level of 5 % with an expected proportion of bacterial growth of 0.09 and the maximal allowable difference of 0.10 non-inferiority limit, which means that a proportional difference of no more than 0.10 in favour of the pre-heated disinfectant was accepted as non-inferiority. To cover potential missing data, the sample size was increased by 16 participants for a total of 220 participants divided into two groups. Due to a non-inferiority hypothesis, the absolute difference of the primary outcome was supplemented with a two-sided 90 % confidence interval (CI); otherwise, a regular two-sided 95 % significance level of 5 % was used.

Bacterial counts and other non-normally distributed variables were analysed with the Mann–Whitney *U* test and normally distributed variables with an unpaired *t*-test. Categorical variables were evaluated with *χ*^2^ -test or Fisher’s exact test as appropriate.

## Results

### Recruitment

**Fig. 1:**
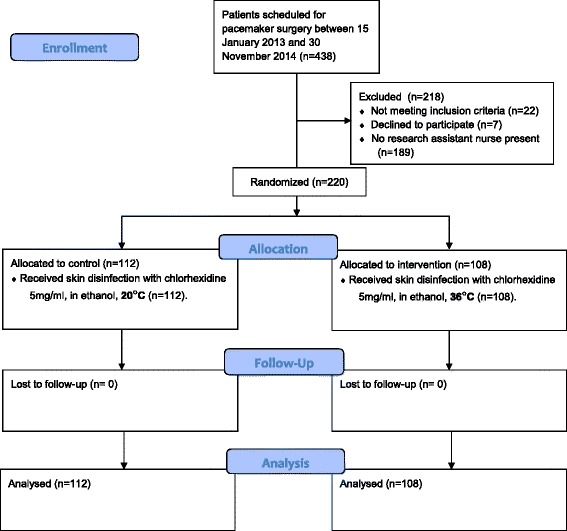
Flow chart

Between January 2013 and November 2014, 220 patients receiving pacemakers were enrolled and randomly allocated to receive skin disinfectant that was preheated (*n* = 108) or at room-temperature (*n* = 112). Patients were followed for three months after surgery (Fig. 1). Characteristics that are known risk factors affecting SSIs are; diabetes, eczema, age and others (Table 1).

**Table 1:** Patients baseline characteristics and surgical factors

Skin disinfection (chlorhexidine 5 mg/mL in 70 % ethanol)	36 °C	20 °C
Characteristics	*N* = 108	*N* = 112
Age (years), mean (SD)	72 (11.9)	74 (12.5)
Body mass index (BMI), mean (SD)	27 (4.5)	27 (5.4)
Length of surgery, minutes, median (IQR)	33 (30)	34 (32)
Colony forming units, median (IQR)	1180 (4690)	2080 (4770)
Male, %	55	57
Eczema, %	6	5
Incision site hair shorten, %	31	26
Diabetes, %	14	18
Bacterial skin growth, %	95	96
Type of surgery		
Device change, %	45.4	46.4
DDD, %	35.2	36.6
VVI, %	6.5	6.3
ICD, %	1.9	3.6
CRT, %	1.9	1.8
Other, %	9.2	5.4

DDD, dual chamber rate adaptive pacemaker. VVI, single ventricular rate adaptive pacemaker. ICD, implantable cardioverter-defibrillator. CRT, cardiac resynchronization therapy. Continuous and dichotomous variables were analysed using *t*-test and Mann Whitney *U* test, no significant differences between groups

Comparison of patients’ characteristics and the surgical factors within each group (disinfectant at 36 °C or 20 °C, respectively)

### Bacterial growth

**Fig. 2:**
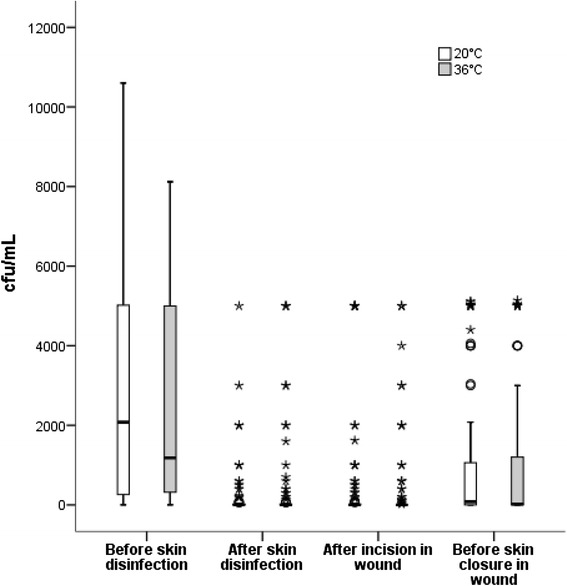
Bacterial growth before and after skin disinfectant. Bacterial growth before and after treatment with skin disinfectant (chlorhexidine 5 mg/mL in 70 % ethanol) at the four time points. The median is identified by a line inside the box. The length of the box is the interquartile range (IQR), and whiskers are min and max if no outliers are present. Outliers of more than 1.5 IQR’s are labeled as (o) and outliers of more than three IQR are labelled as (*)

One hundred and one of 106 (95.3 %) skin cultures taken before receipt of preheated skin disinfectant showed growth compared with 108 of 112 (96.4 %) taken before receipt of room temperature disinfectant. Thirty of 105 (28.6 %) skin cultures after receipt of preheated skin disinfectant showed growth compared with 32 of 112 (28.6 %) receiving room-temperature skin disinfectant. The absolute difference in growth was zero (90 % CI −10.1 to 10.1) (Table 2). Microorganisms identified before skin disinfection included *Propionibacterium acnes*, coagulase-negative staphylococci (CoNS), alpha-haemolytic streptococci, anaerobic diphtheriod rods, *Bacillus* species, *Micrococcus* species, *Staphylococcus aureus*, anaerobic gram-positive cocci, *Proteus* species, and *Rothia mucilaginosa*. At subsequent time points, the amount of different bacteria and the number of bacteria changed (Fig. 2). The most frequently identified pathogen after skin disinfection both on the skin and in the wound was *P. acnes* followed by CoNS (Table 3). No significant differences were observed in any of the cultures at the 4 time-points regarding growth or median or mean cfu/mL between the groups (Table 4).

**Table 2:** Bacterial growth at the four time points

Skin disinfection (chlorhexidine 5 mg/mL in 70 % ethanol)	All			Men		Women	
	36 °C	20 °C	Absolute difference	36 °C	20 °C	36 °C	20 °C
	*n* = 106	*n* = 112	(90 % CI)	*n* = 62	*n* = 62	*n* = 44	*n* = 50
Before skin disinfection	95.3	96.4		100	100	88.6	92
After skin disinfection^a^	28.6	28.6	0 (−0.101 to 0.101)	40.3	43.5	11.6	10
After incision (wound)	24.5	30.4	−0.059 (−0,158 to 0.040)	40.3	45.2	2.3	12
Before wound closure (wound)	53.8	62.5	−0.087 (−0,197 to 0,023)	74.2	82.3	25	38

Proportion of swabs from patients that showed any bacterial growth at the various time points. Data are shown as percentages and absolute difference with confidence intervals (CI)

^a^Primary outcome

**Table 3:** Species of bacteria identified

	Culture 1		Culture 2		Culture 3		Culture 4	
Microorganism	36 °C	20 °C	36 °C	20 °C	36 °C	20 °C	36 °C	20 °C
	*n* = 106	*n* = 112	*n* = 105	*n* = 112	*n* = 106	*n* = 112	*n* = 106	*n* = 112
P. acnes	83	86	25	25	22	29	47	52
CoNS	83	90	2	4	2	6	23	29
S. aureus	2	2	1	1	-	-	-	-
Alpha-haemolytic	9	9	-	-	-	-	-	1
Anaerobic diphtheriod rods	5	3	-	1	1	1	1	1
Bacillus species	2	1	-	-	-	-	-	-
Micrococci	4	-	-	-	-	-	-	-
Anaerobic gram-positive cocci	1	2	-	1	1	-	-	3
Proteus sp.	2	-	-	-	-	-	1	-
R. mucilaginosa	-	1	-	-	-	-	-	-

No significant differences between study groups for any microorganism at any time point. Chi-2 test were used as statistical method

Bacterial species identified from swabs taken from patients who received preheated and room-temperature skin disinfectant (chlorhexidine 5 mg/mL in 70 % ethanol) with growth in cultures taken perioperatively; 1) before skin disinfection, 2) after skin disinfection, 3) after incision (wound), and 4) before skin closure (wound)

**Table 4:** Mean bacterial growth

	Mean cfu/mL					
Skin disinfection (Chlorhexine 5 mg/mL in 70 % ethanol)	All		Men		Women	
	36 °C	20 °C	36 °C	20 °C	36 °C	20 °C
	*n* = 106	*n* = 112	*n* = 62	*n* = 62	*n* = 44	*n* = 50
Before skin disinfection	2388	2687	3516	3734	825	1389
After skin disinfection	298	199	500	277	5	85
After incision (wound)	347	383	592	576	1	144
Before wound closure (wound)	973	1004	1570	1615	131	258

No statistically significant differences between preheated and room-temperature skin disinfectant within study groups at any time point evaluated for all patients as well as for men and women separately. Mann Whitney *U* test used as statistical method. Cultures showed that male had significantly more bacteria at all four time-points than females irrespectively of temperature. Mann Whitney *U* test used as statistical method

Bacterial growth, represented as mean cfu/mL, observed in cultures from patients who received preheated skin disinfectant compared with room-temperature skin disinfectant at the four perioperative time-points. Data were combined for the overall group and for males and females separately

### Gender

Cultures showed that males had significantly more bacteria at the four time-points than females irrespectively of the temperature of the skin disinfectant. Gender differences at the first time-point appeared in both the preheated (*p* = 0.011) and room-temperature disinfectant groups (*p* = 0.037). A gender difference was also seen during the second, third, and fourth time-point (*p* ≤ 0.001). Analyses performed on the overall group or with males and females separately showed no significant differences regarding disinfection with preheated or room-temperature disinfectant (Table 4).

### Surgical site infections

There were no significant differences in SSIs three months postoperatively between patients who received preheated versus room temperature skin disinfectant; 1 (female) of 108 (0.9 %) vs 2 (1 male and 1 female) of 112 (1.8 %), respectively. At the time of surgery, samples from females showed growth only before skin disinfection, whereas the male displayed growth of *P. acnes* at all four time-points and CoNS at the first and last time points. Cultures taken postoperatively, when patients were diagnosed for SSI, were negative for both female patients, whereas the cultures from the male patient were positive for *S. aureus*, CoNS, *P. acnes*, and beta-haemolytic streptococci group G.

## Discussion

### Bacterial growth

In this study, no significant differences were found related to the presence of bacteria, confirming that preheated and room-temperature skin disinfectant have similar bactericidal effects, as shown in our previous pilot study [18]. The results clearly show that preheated or room temperature skin disinfectant reduce the number of bacteria on the skin and prevent SSIs equally well. The most frequently identified bacteria in the wound after disinfection was *P. acnes*, which also inhabits deeper layers of the skin [19, 20]. A possible explanation is that when the incision is made, deeper layers of the skin are exposed and *P. acnes* relocate into the wound [20]. *P. acnes* can be a causative factor of SSIs [20–23].

### Gender

Earlier studies have shown that the amount of bacteria differs between males and females and this study supports that finding [12, 24]. The effectiveness of preheated or room-temperature skin disinfectant was equivalent.

### Surgical site infections

The male patient who experienced SSI showed growth in cultures taken at all four time-points. Two other species of bacteria, *S. aureus* and beta-haemolytic streptococci group G, were also found when the SSI was diagnosed. These species were not present at the time of surgery. Cultures taken postoperatively to determine the causative pathogens were negative in the female patients with SSIs. According to the criteria, SSI could be diagnosed as purulent drainage, fever, tenderness, and usually a positive culture or diagnosed as SSI by the attending physician [1]. The reason these cultures did not show any growth could possibly be due to an ability of the bacteria to protect themselves with biofilm [19], but also because *P. acnes* has a slow-growing nature [23].

### Limitations

There are limitations to this study. First, this study had a power problem related to the population size because the power calculation was made based on 10 % growth, whereas in the present study the patients showed 28.6 % bacterial growth after skin disinfection. Secondly, this study was designed as a non-inferiority trial to detect differences in bacterial contamination, not to detect differences in SSIs.

In conclusion, recommendations aimed at preventing SSIs should be evidence based [25]. The assumption that preheated skin disinfection is non-inferior to room-temperature disinfectant in bacterial reduction appears to be correct. We therefore suggest that preheated skin disinfection can be used routinely prior to clean surgery. Additional studies involving other types of surgery, including those affecting other body sites and levels of complexity and length, are warranted.
